# Exploring the Relationship between Perfectionism and Pain in Patients with Temporomandibular Disorders: A Cross-Sectional Study

**DOI:** 10.1155/2023/2857115

**Published:** 2023-05-31

**Authors:** Xin Xiong, Shi-Yong Zhang, Jing Zhang, Nan Jiang, Li-Ming Zhang, Hao-Lun Yang, Yuan Yue

**Affiliations:** ^1^National Clinical Research Center for Oral Diseases, State Key Laboratory of Oral Diseases, Department of Orthodontics, West China Hospital of Stomatology, Sichuan University, Chengdu, Sichuan, China; ^2^Department of Temporomandibular Joint, West China Hospital of Stomatology, Sichuan University, Chengdu, Sichuan, China; ^3^Department of Nursing, West China Hospital of Stomatology, Sichuan University, Chengdu, Sichuan, China; ^4^National Clinical Research Center for Oral Diseases, State Key Laboratory of Oral Diseases, Department of Oral & Maxillofacial Surgery, West China Hospital of Stomatology, Sichuan University, Chengdu, China; ^5^Rehabilitation Medicine Center, Department of Rehabilitation Medicine, West China Hospital, Sichuan University, Chengdu, Sichuan, China; ^6^Department of Prosthodontics, West China Hospital of Stomatology, Sichuan University, Chengdu, Sichuan, China

## Abstract

**Objectives:**

The purpose of this cross-sectional study was to examine the relationship between perfectionism and pain in patients with temporomandibular disorders (TMDs).

**Methods:**

A total of 345 TMD patients were included. A questionnaire consisting of questions of demographic information, the 15-item short form of the Hewitt and Flett Multidimensional Perfectionism Scale, and the Patient Health Questionnaire-4 (PHQ-4) was distributed. According to the diagnostic criteria for TMDs, patients were categorized as pain-related (PT) and non-pain-related (NPT) groups, whereas PT patients were further divided into patients with pain-related TMDs only (OPT) and patients with combined pain-related and intra-articular TMDs (CPT). Data were analyzed using the chi-square test, Spearman's correlation, and logistic regression analysis with the significance level set at *p* < 0.05.

**Results:**

There were 68 patients in the NPT group, 80 in the OPT group, and 197 in the CPT group. PT patients had significantly higher perfectionism scores (63.58 ± 13.63) than NPT patients (56.32 ± 12.95, *p* < 0.001). The PHQ-4 score in the PT group was also higher. After adjusting the PHQ-4 scores, perfectionism scores of the PT group were 6.11 points higher than those in the NPT group (*p* < 0.001). There were no statistical differences in all parameters of OPT and CPT groups (*p* > 0.05). Perfectionism in total, other-oriented perfectionism (OOP), and socially prescribed perfectionism (SPP) showed significant but weak correlations with PHQ-4 scores (*p* < 0.001), while self-oriented perfectionism (SOP) was also significantly but very weakly correlated with PHQ-4 scores (*p* < 0.05).

**Conclusions:**

Pain-related TMD patients exhibited higher perfectionism scores than NPT patients, and neither their perfectionism nor pain scores were correlated with intra-articular diseases of TMJ. OOP and SOP presented weak correlations with psychological distress in TMD patients. It is suggested that pain-related TMD patients could be screened for perfectionism and perfectionism could be considered when proposing psychological treatment strategies to PT patients.

## 1. Introduction

Temporomandibular disorders (TMDs) are defined as a set of orofacial conditions related to abnormalities in the structure, function, or physiology of the temporomandibular joint (TMJ) and masticatory muscles which may be associated with other systemic and comorbid medical conditions [1]. Study has revealed that TMDs are the leading causes of chronic pain in the temporomandibular region, with a prevalence in the general population at 10–31% [2–4]. Moreover, TMDs, particularly pain-related TMDs, are widely recognized for impairing both oral health-related and overall quality of life [5, 6].

The etiology of TMDs is generally believed to be multifactorial, which comprises trauma, stress, parafunctional habits, and psychological and genetic factors, embedded in the biopsychosocial model [7–9]. The bidirectional relationship between TMDs and psychological diseases is well reported. Psychological factors including stress, anxiety, and depression might increase the risk of developing TMDs [10]; correspondingly, prolonged TMD symptoms could aggravate psychological status of patients [11, 12]. Hence, investigations on the psychological characteristics of TMD patients would benefit both diagnosis and treatment approaches for TMDs.

The prominent definitions of perfectionism are the setting of excessively high standards on performance and self-criticism over not meeting standards [13, 14]. Perfectionism has two major dimensions: perfectionistic strivings and perfectionistic concerns [15]. The former reflects the adaptive facet of perfectionism, such as positive effect, and the latter indicates the maladaptive facet, such as negative affect, depression, stress, and anxiety [14]. According to the model proposed by Hewitt and Flett, perfectionism has inter-personal and intra-personal aspects [16]. In the model, self-oriented perfectionism (SOP), which is a key concept of perfectionistic strivings, is defined as the tendency to set high standards for oneself while striving for meeting those standards. Other-oriented perfectionism (OOP), as the other key element of perfectionistic strivings, is the expectation for significant others to achieve unrealistic standards [14]. Socially prescribed perfectionism (SPP), which stands for perfectionistic concerns, is the perception that other people hold exceedingly high standards for oneself with continuous evaluation for his/her achievement. Some research studies suggest that SOP and OOP have both maladaptive and adaptive characteristics, while SPP is primarily maladaptive due to its strong association with negative traits and psychological distress [17].

Previous studies have reported the association between perfectionism and diseases such as eating disorder, sleep disorder, and chronic pain. More specifically, perfectionism could affect individuals' pain catastrophizing and pain-related fear. However, to the best of our knowledge, no prior research has examined links between perfectionism and temporomandibular disorders, which is a common source of chronic orofacial pain.

Thus, this study aims to explore the perfectionism of patients with temporomandibular disorders. A subsequent goal is to analyze the correlations between the perfectionism and psychological state of TMD patients. The null hypothesis was proposed that there were no differences in perfectionism between patients with or without pain-related TMDs.

## 2. Materials and Methods

The ethical approval for this retrospective study was obtained from the Institutional Review Board of West China Hospital of Stomatology (no. WCHSIRB-CT-2022-241).

### 2.1. Participants

*G* ^*∗*^power (*G*^*∗*^Power Team, Belgium) was used to calculate the sample size. According to our pilot study, the significance level was set as 0.05, level of *β* was set as 0.05 (95% power), effect size was set as 0.5, and allocation ratio was assumed as 4 : 1 (painful TMD to non-painful TMD). This calculation yielded a minimum sample size of 328 patients.

Consecutive patients (≥12 years) seeking care at the Department of TMJ were recruited over a 6-month period. Eligible patients signed the informed consent when filling the questionnaires. The exclusion criteria were as follows: (1) presence of major psychiatric disorders and/or drug abuse; (2) presence of major trauma and/or operations; (3) presence of major autoimmune and/or metabolic diseases; (4) current consumption of central nervous system agents; and (5) cognitive impairment and/or illiteracy. All patients underwent a standardized examination according to Axis I of Diagnostic Criteria for Temporomandibular Disorders (DC/TMD) [18].

According to the DC/TMD diagnostic results, the patients were classified into two groups: the PT group, patients with pain-related TMDs, and the NPT group, patients with non-painful intra-articular TMDs, while the PT group was further subgrouped into two groups: patients with pain-related TMDs only (OPT group) and patients with combined pain-related and intra-articular TMDs (CPT group).

### 2.2. Data Collection

A visual analog scale ranging from 0 to 10 was used to measure the current pain intensity (VAS-c) around the TMJ area and the worst pain intensity in the last month (VAS-m).

A questionnaire designed to assess the perfectionism and general psychological status of the patients consists of three parts. Part one is about the demographic characteristics, including sex, age, education level, and family per capita monthly income.

The second part is the 15-item short form of the Hewitt and Flett Multidimensional Perfectionism Scale, which involves 5 items about self-oriented perfectionism, 5 items about other-oriented perfectionism, and 5 items about socially prescribed perfectionism, respectively [19].

The third part is the Patient Health Questionnaire-4 (PHQ-4) [20], which has been used in our previous studies [5, 21]. A 4-point Likert scale ranging from 0 (not at all) to 3 (nearly every day) is used. The total possible score of PHQ-4 ranges from 0 to 12; higher scores indicate greater severity. A score of more than 2 points on Patient Health Questionnaire-2 (PHQ-2) and Generalized Anxiety Disorder-2 (GAD-2) subscale represents the presence of depression and anxiety. The PHQ-4 was validated among Chinese adults with satisfactory reliability and validity [22].

### 2.3. Statistical Analysis

Cronbach's *α* coefficient was used as the reliability index for the questionnaires. The inter-observer and intra-observer agreement of the VAS was assessed using the intra-class correlation coefficient (ICC).

In the case of continuous variables, the *t*-test or Mann–Whitney *U* test was used. The Pearson chi-square test or Fisher's exact test was used for categorical variables. Multiple linear regression models were practiced for the scores of perfectionism, which was considered as continuous dependent variable [21]. Correlation analysis was performed using Spearman analysis. A moderate correlation is defined as 0.6  >  *r* ≥ 0.4, a weak correlation as 0.4  >  *r* ≥ 0.2, and very correlation as *r* < 0.2. All statistical analyses were performed with the R package (https://www.R-project.org, the R Foundation) and Empowerstats (X&Y Solutions, Inc., Boston). An *α* level of 0.05 was considered statistically significant.

## 3. Results

The sample was composed of 345 subjects, resulting in two main groups: NPT group (*n* = 68) and PT group (*n* = 277). No statistical differences were shown in demographic characteristics between the two groups (*p* > 0.05, Table 1).

Cronbach's *α* coefficient of the total questionnaire of perfectionism is 0.92, and Cronbach's *α* coefficient of SOP is 0.85, that of OOP is 0.80, and that of SPP is 0.84, indicating good internal consistency. The inter-observer ICC of the VAS was 0.921, and the intra-observer ICCs were 0.935 and 0.928.

The total scores of perfectionism were 56.32 ± 12.95 in the NPT group and 63.58 ± 13.63 in the PT group, respectively. There were statistical differences in the scores of GAD-2, the three subscales of perfectionism (SOP, OOP, and SPP), and the total scores of perfectionism between two groups (Table 2, *p* < 0.05).

Analysis of the subgroups of PT patients demonstrated no statistical differences in all the parameters between the OPT and CPT groups (*p* > 0.05, Table 3).

**Figure 1:**
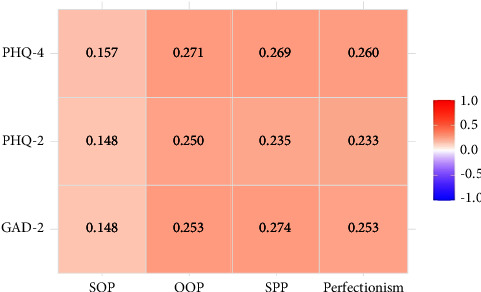
Correlation between perfectionism and psychological status. PHQ-4, Patient Health Questionnaire-4; GAD-2, Generalized Anxiety Disorder-2.

Correlation analysis showed that perfectionism in total, OOP, and SPP had significant but weak correlation with PHQ-4 scores (*p* < 0.001), while SOP had significant but very weak correlation with GAD-2, PHQ-2, and PHQ-4 scores (*p* < 0.05, Figure 1). Multiple linear regression analysis indicated that the PT group had higher perfectionism scores (β = 7.25, 957% CI: 3.67–10.84). After adjusting the PHQ-4 total scores, the effect was still significant (β = 6.11, 95% CI: 2.667–9.57) (Table 4).

## 4. Discussion

In this study, we proposed to investigate the perfectionism of TMD patients as well as its association with the patients' psychological state. The findings suggested that painful TMD patients (PT group) exhibited higher perfectionism scores than the non-painful ones (NPT group). The PT group still exhibited higher perfectionism scores even with psychological scores adjusted. In addition, the study supported that perfectionism was weakly correlated with psychological status among patients. The null hypothesis we posed that there were no differences in perfectionism between the painful and non-painful TMD patients had also been proven to be valid.

Cao et al. [23] and Yap et al. [24] reported relatively low proportion (59.03% and 52.54%) of pain-related TMDs in Chinese TMD patients, while in our study, around 4/5 of the sample were PT patients, similar with a cross-sectional study in Portugal [25]. One possible reason for that might be the difference in the methods of recruitment because people would actively seek treatments for pain syndromes but lack the initiative to go to a clinic when having intra-articular conditions. Besides, we detected “pain” by asking patients whether they have discomfort or other unpleasant experience associated with the temporomandibular joint, masticatory muscles, or the adjacent structures referring to the revised definition of pain [26], which might increase the detection rate of pain conditions.

Lombardo et al. reported that the mean scores of self-oriented perfectionism among obsessive-compulsive disorder patients were 21.50 ± 7.57, those of other-oriented perfectionism were 19.15 ± 5.94, and those of socially prescribed perfectionism were 17.58 ± 6.71, respectively [27], which were relatively lower in comparison to the scores obtained among the TMDs patients in our study. Social demographic factors such as culture background age, gender, race, and income might contribute to the differences in scores of perfectionism. Chinese people embrace the culture of hard work, motivating them to focus more on competition, outcomes, and success than quality of life, which can consequently account for their higher perfectionism scores. Few studies have performed the short form of the Hewitt and Flett Multidimensional Perfectionism Scale to evaluate perfectionism in Chinese population; therefore, researchers could perform the scale more often for evaluations in related studies in the future.

Compared with individuals without pain-related TMDs, those with PT had higher levels of psychological distress and worse temporomandibular-related quality of life, especially in the psychological domains. However, whether PT patients had intra-articular diseases was not associated with the psychological distress [28]. Our study found that there was no statistical difference in perfectionism, VAS, and other variables between OPT and CPT patients, indicating that perfectionism was not significantly associated with the intra-articular diseases, which is concordant with previous studies [29]. It is common that the radiographic findings do not significantly correlate with clinical pain symptoms [30, 31]. These findings further approved that the way an individual feels pain is influenced by cognitive, emotional, and behavioral factors.

The biopsychosocial model posits that pain, especially chronic pain, is the result of a dynamic integration of biological processes, psychological factors, and sociocultural contexts. Emotional, cognitive, and behavioral factors could contribute to the patients' pain experience. Previous research highlights a bidirectional relationship between pain and emotional distress, in which pain is predicted by psychological struggles and psychological struggles are influenced by pain [32]. Considering that perfectionism is associated with higher psychological distress [33], pain-related TMD patients may be more susceptible to negative consequences. Sheila et al. reported that, in women with fibromyalgia, maladaptive perfectionism is associated with activity avoidance regardless of the degree of severity of the disease and the pain experienced in the situation, reducing the patients' ability to work and participate in social activities [34]. Therefore, perfectionism might be a target when cognitive behavioral therapy is undergone to manage pain in TMD patients.

A survey recruiting 817 participants showed that OOP and SPP were indirectly related to dysfunctional eating behaviors through the mediation of psychological distress, whereas non-significant mediation result was found for SOP [35]. A longitudinal study reported that OOP and SPP were associated with lower posttreatment reduction in depression over treatment in psychiatric patients receiving cognitive behavioral therapy [36]. The correlation analysis in our study also showed that OOP and SPP had relatively closer correlations with psychologic distress than SOP. Combing the results and previous studies, we further confirmed the maladaptive nature of OOP and SPP. Hence, further research efforts should be directed to the investigation of the roles of OOP and SPP in the psychological dimension of TMDs.

This is a pioneer study that comprehensively investigated the correlation between perfectionism and TMD pain. TMD patients with painful syndromes had a higher level of perfectionism. Thus, our study provides reference to future studies concerning the role of perfectionism on the etiology and treatments of pain-related TMDs. Besides, our study further evaluated the subdimensions of perfectionism, which preliminarily confirmed the important associations of OOP and SPP with psychological problems. As already mentioned above, SPP, also conceptualized as a perfectionistic concern, has a strong link with psychological distress like depression, stress, and anxiety.

Nonetheless, our study had several limitations. The cross-sectional study has limitations in permitting casual relationships between pain, psychological distress, and perfectionism. Therefore, future prospective and longitudinal research designs are required to establish causality. Despite the satisfactory reliability and validity of PHQ-4 scale for the assessment of anxiety and depression in TMD patients [37], PHQ-4 scale is a comparatively simple measurement with relatively limited items and aspects about the psychological status and personality of individuals. Additionally, there was a lack of healthy controls included for comparison; therefore, whether perfectionism in TMD patients or NPT patients is higher than that in TMD-free individuals is unknown. Age- and sex-matched TMD-free individuals could be set as controls in future studies.

## 5. Conclusion

In TMD patients, the painful individuals exhibited higher perfectionism scores than the non-painful ones, and OOP and SPP were weakly correlated with psychological distress in TMD patients.

## Figures and Tables

**Table 1:** Demographic data of the participants.

	NPT group (*n* = 68)	PT group (*n* = 277)	*p* value
Age	25.19 ± 7.57	26.59 ± 8.05	0.171
Sex			
Male	13 (19.12%)	41 (14.80%)	0.380
Female	55 (80.88%)	236 (85.20%)	
Education level			0.312
High school or below	16 (23.53%)	55 (19.86%)	
College	41 (60.29%)	192 (69.31%)	
Above college	11 (16.18%)	30 (10.83%)	
Family per capita monthly income			0.229
Less than 3000 yuan	6 (8.82%)	32 (11.55%)	
3000∼600 yuan	34 (50.00%)	107 (38.63%)	
More than 6000 yuan	28 (41.18%)	138 (49.82%)	

**Table 2:** Difference in VAS, psychological status, and perfectionism scores between the NPT and PT groups.

	NPT group (*n* = 68)	PT group (*n* = 277)	Total (*n* = 345)
VAS-c	0.00 (0.00–1.00)	2.00 (1.00–3.00)	2.03 ± 1.75
VAS-m	0.00 (0.00–1.25)	3.00 (1.00–4.00)	2.51 ± 2.13
GAD-2	1.24 ± 1.26	1.71 ± 1.61	1.61 ± 1.55
PHQ-2	1.38 ± 1.46	1.62 ± 1.51	1.57 ± 1.50
PHQ-4	2.62 ± 2.56	3.32 ± 2.92	3.19 ± 2.87
Self-oriented perfectionism	20.00 ± 5.06	22.13 ± 4.94	21.71 ± 5.03
Other-oriented perfectionism	18.54 ± 4.51	20.81 ± 4.96	20.37 ± 4.95
Socially prescribed perfectionism	17.78 ± 4.76	20.64 ± 5.43	20.08 ± 5.42
Total scores of perfectionism	56.32 ± 12.95	63.58 ± 13.63	62.15 ± 13.79

Data are presented as mean ± standard deviation or median (Q1, Q3).

**Table 3:** Difference in demographic parameters, psychological status, VAS, PHQ-4, and perfectionism scores between the OPT and CPT groups.

	OPT group (*n* = 80)	CPT group (*n* = 197)	*p* value
Age	26.98 ± 8.64	26.43 ± 7.81	0.611
Sex			
Male	9 (11.25%)	32 (16.24%)	0.289
Female	71 (88.75%)	165 (83.76%)	
VAS-c	2.00 (1.00–3.00)	2.00 (1.00–3.00)	0.611
VAS-m	3.00 (2.00–4.00)	3.00 (1.00–4.00)	0.288
GAD-2	1.73 ± 1.53	1.70 ± 1.64	0.909
PHQ-2	1.45 ± 1.35	1.69 ± 1.57	0.241
PHQ-4	3.17 ± 2.65	3.39 ± 3.03	0.857
Self-oriented perfectionism	22.51 ± 4.65	21.97 ± 5.06	0.408
Other-oriented perfectionism	21.25 ± 4.67	20.63 ± 5.08	0.350
Socially prescribed perfectionism	20.93 ± 5.36	20.52 ± 5.46	0.577
Total scores of perfectionism	64.69 ± 13.22	63.13 ± 13.80	0.389

Data are presented as mean ± standard deviation or median (Q1, Q3).

**Table 4:** Multiple linear regression models for the association between perfectionism and pain-related temporomandibular disorders.

	Non-adjusted	Adjust I
NPT group	Reference	Reference
PT group	7.25 (3.67, 10.84) *p* < 0.001	6.11 (2.66, 9.57) *p* < 0.001

The non-adjusted model adjusts for: none. Adjust I model adjusts for: the total scores of Patient Health Questionnaire-4.

## Data Availability

The data used to support the findings of this study are available from the corresponding author upon reasonable request.
